# Synthesis, Biological, and Computational Evaluation of Antagonistic, Chiral Hydrobenzoin Esters of Arecaidine Targeting mAChR M1

**DOI:** 10.3390/ph13120437

**Published:** 2020-11-30

**Authors:** Marius Ozenil, Jonas Aronow, Daniela Piljak, Chrysoula Vraka, Wolfgang Holzer, Helmut Spreitzer, Wolfgang Wadsak, Marcus Hacker, Verena Pichler

**Affiliations:** 1 Department of Biomedical Imaging and Image-guided Therapy, Division of Nuclear Medicine, Medical University of Vienna, 1090 Vienna, Austria; marius.ozenil@meduniwien.ac.at (M.O.); jonas.aronow@meduniwien.ac.at (J.A.); daniela.piljak@gmx.at (D.P.); chrysoula.vraka@meduniwien.ac.at (C.V.); wolfgang.wadsak@meduniwien.ac.at (W.W.); marcus.hacker@meduniwien.ac.at (M.H.); 2 Department of Pharmaceutical Chemistry, Faculty of Life Sciences, University of Vienna, 1090 Vienna, Austria; wolfgang.holzer@univie.ac.at (W.H.); helmut.spreitzer@univie.ac.at (H.S.); 3 CBmed GmbH—Center for Biomarker Research in Medicine, 8036 Graz, Austria

**Keywords:** muscarinic, drug development, subtype selectivity

## Abstract

Muscarinic acetylcholine receptors (mAChRs) are a pivotal constituent of the central and peripheral nervous system. Yet, therapeutic and diagnostic applications thereof are hampered by the lack of subtype selective ligands. Within this work, we synthesized and chemically characterized three different stereoisomers of hydrobenzoin esters of arecaidine by NMR, HR-MS, chiral chromatography, and HPLC-logP. All compounds are structurally eligible for carbon-11 labeling and show appropriate stability in Dulbecco’s phosphate-buffered saline (DPBS) and F12 cell culture medium. A competitive radioligand binding assay on Chinese hamster ovary cell membranes comprising the human mAChR subtypes M1-M5 showed the highest orthosteric binding affinity for subtype M1 and a strong influence of stereochemistry on binding affinity, which corresponds to in silico molecular docking experiments. K_i_ values toward M1 were determined as 99 ± 19 nM, 800 ± 200 nM, and 380 ± 90 nM for the (*R*,*R*)-, (*S*,*S*)-, and racemic (*R*,*S*)-stereoisomer, respectively, highlighting the importance of stereochemical variations in mAChR ligand development. All three stereoisomers were shown to act as antagonists toward mAChR M1 using a Fluo-4 calcium efflux assay. With respect to future positron emission tomography (PET) tracer development, the (*R*,*R*)-isomer appears especially promising as a lead structure due to its highest subtype selectivity and lowest K_i_ value.

## 1. Introduction

Muscarinic acetylcholine receptors (mAChRs) are G protein-coupled receptors, which are strongly involved in the parasympathetic nervous system’s signal transduction. mAChRs appear as five subtypes, which differ in their expression pattern and downstream signaling [1]. However, their conserved orthosteric binding units hamper subtype selective molecular targeting [2]. Current anticholinergic drugs with market approval are mainly antagonists or inverse agonists [3]. Regarding therapeutic applications, mAChR agonists are in clinical use for the treatment of glaucoma [4], Sjögren’s syndrome [4], and underactive bladder [5]. mAChR agonists have also been proposed as promising drugs for the treatment of Alzheimer’s disease symptoms, hitherto, no compound passed clinical trial phase 3 [6]. Several mAChR antagonists targeting primarily the M1 subtype are used in medicine mainly for the treatment of Parkinson’s disease [7] and peptic ulcers [8]. However, the clinical applicability of mAChR agonists and antagonists is often restrained by side effects related to the activation or inhibition of other mAChR subtypes [7]. Considering the insufficient penetration of the blood–brain barrier as well as unsuitable pharmacokinetics and -dynamics in treatment regimens of e.g., Parkinson’s disease, these drugs often merely have peripheral effects on disease symptoms. Hence, as they do not act on the disease origin [9], they do not have the ability to alter disease progression. Therefore, clinical management is solely based on symptomatic treatment.

The pivotal role of mAChRs in human physiology and their involvement in neuropathogenesis is well known, yet the underlying molecular mechanisms remain poorly understood [2]. A suitable positron emission tomography (PET) tracer targeting the mAChRs would allow for in vivo molecular imaging of mAChRs and hence strongly contribute to increase the knowledge in mAChR research. The basic understanding of the molecular mechanism is the only guide to establish personalized precision medicine based on image-guided therapy applications for central nervous system (CNS) diseases. Irrespective of numerous efforts, so far, no mAChR PET radiotracer has found widespread clinical application. Predominantly, this can be attributed to unsuccessful attempts to meet all the stringent requirements such as high affinity (ca. 3–50 nM) [10], subtype selectivity, metabolic stability, in vivo specific binding, and many more [11]. The requirements and parameters defining an effective and safe medication are highly dependent on the purpose (therapy or diagnosis) and the site of action (central or peripheral targets) and have to be considered already during the drug discovery process. In contrast to imaging agents, therapeutic CNS drugs allow less stringent attributes in terms of structure and binding affinity (µM to nM), but toxicology, functionality profile, and functional subtype selectivity are more in the spotlight [9,12].

**Figure 1:**
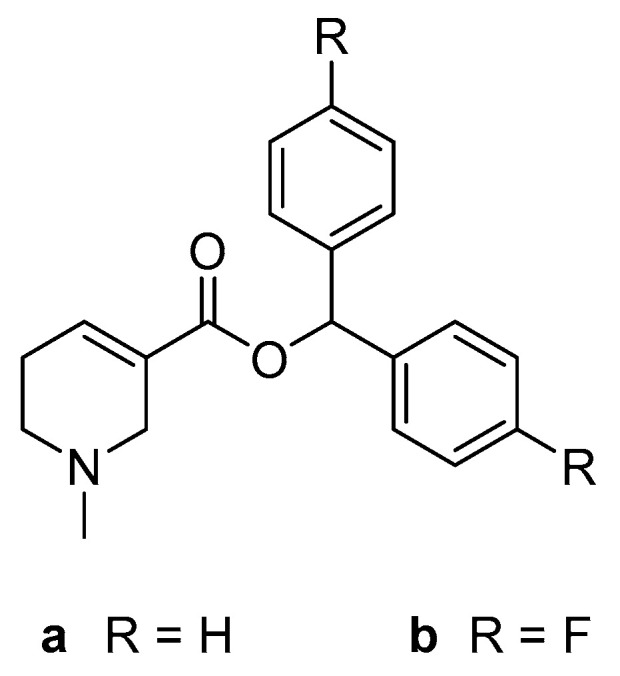
General structure of previously studied diphenylmethyl esters of arecaidine [13].

Concluding, the repertoire of mAChR ligands for both therapy and diagnosis is characterized by a lack of high affinity and simultaneously subtype selective compounds. Diphenylmethyl esters of arecaidine (**a** and **b**, Figure 1) were recently discovered as orthosteric mAChR ligands with low nanomolar affinity and pronounced M1 selectivity. Unfortunately, strong nonspecific binding observed in the preclinical evaluation restrained their use as PET tracers [13]. Considering that nonspecific binding is often caused by hydrophobic interactions [14], this work aims to synthesize and evaluate similar compounds with reduced lipophilicity. In this study, we introduce the synthesis of chiral hydrobenzoin esters of arecaidine, which are fully characterized by nuclear magnetic resonance (NMR) spectroscopy and high resolution-mass spectrometry (HR-MS), and the purity was assessed by chiral and reversed phase-high performance liquid chromatography (HPLC). Physicochemical parameters as well as stability, affinity, and functionality toward mAChRs were assessed by in vitro methods for all synthesized compounds. In order to understand the difference in binding affinities, the binding poses of the novel compounds were assessed by in silico docking studies.

## 2. Results and Discussion

### 2.1. Chemistry

**Figure 2:**
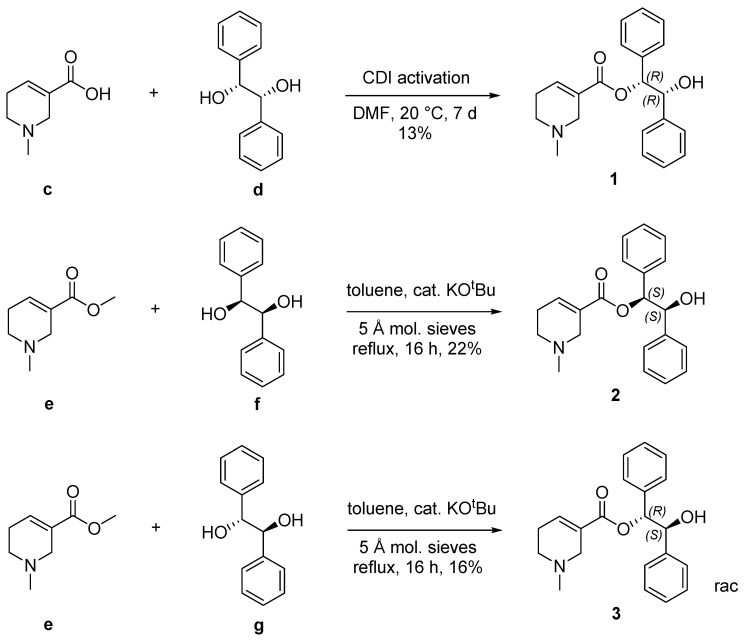
Stereoselective synthesis of hydrobenzoin esters of arecaidine.

Hydrobenzoin esters of arecaidine were prepared and characterized by NMR spectroscopy, HR-MS, logP, RP-HPLC, and chiral HPLC. The esterification of (*R*,*R*)- and (*S*,*S*)-hydrobenzoin (**d**, **f**) yielded the enantiomerically pure esters **1** and **2**, whereas esterification with *meso*-hydrobenzoin (**g**) yielded racemic **3** (Figure 2). Previously, arecaidine esters were prepared in low yields via 1,1′-carbonyldiimidazole (CDI)-mediated esterification [13]. Within this work, we describe a transesterification procedure from the readily available arecaidine methyl ester (**e**, arecoline). Regarding the synthesis of hydrobenzoin esters, the transesterification (synthesis of **2**) is characterized by a 70% increased yield and less experimental effort compared to the CDI-mediated esterification (synthesis of **1**). The vicinal coupling constant of the hydrobenzoin part is the most notable difference in NMR spectra between **1** or **2** (7.2 Hz) to **3** (5.8 Hz). Chiral HPLC confirmed that no racemization occurred during both methods (Supporting Information).

### 2.2. Evaluation of Physico-Chemical Parameters and Stability

HPLC-logP_ow_^pH7.4^ values were found to be 2.24 ± 0.12 (**1**=**2**) and 2.39 ± 0.10 (**3**), which are much lower compared to arecaidine diphenylmethyl ester (3.32 ± 0.04) [13]. Consequently, **1**, **2**, and **3** are expected to be less prone to nonspecific binding. The predictive power of logP for blood–brain barrier permeability is controversially discussed, and several desirable logP ranges were postulated. However, the HPLC-logP_ow_^pH7.4^ values of **1**, **2**, and **3** comply with every single favorable logP range compared recently [15]. In addition, other chemical characteristics of **1**, **2**, and **3**, such as molecular weight (337.42 g/mol), number of hydrogen bonds (3 acceptors, 1 donor), polar surface area (49.8 Å^2^), rotatable bond count (6), and possession of a tertiary nitrogen with a positive charge at pH 7–8 (calculated pKa = 7.8) are in good accordance with desired properties for CNS active drugs [16]. Furthermore, analysis by SwissADME [17] indicates that **1**, **2**, and **3** are no P-glycoprotein substrates and no PAINS [18] or Brenk [19] alerts were caused.

**Figure 3:**
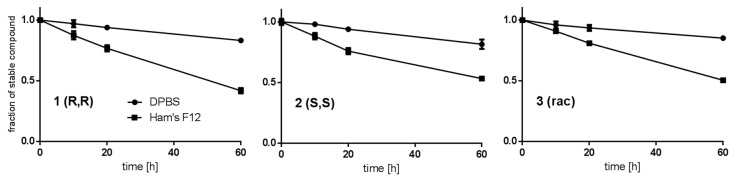
Stability of **1**, **2,** and **3** in Dulbecco’s phosphate-buffered saline (DPBS) and Ham’s F12 cell culture medium at 37 °C. Error bars represent the standard deviation.

The stability of **1**, **2**, and **3** in buffer and cell culture medium was investigated because their disintegration could impair biological testing. **1**, **2**, and **3** show a comparable rate of decomposition in Dulbecco’s phosphate-buffered saline (DPBS) and Ham’s F12 cell culture medium at 37 °C over a time period of 60 h (Figure 3), which is slow enough to allow for biological testing and drug development. For **1**, **2**, and **3**, the decomposition is faster in Ham’s F12 cell culture medium compared to DPBS. After 60 h in DPBS **1**, **2**, and **3** were decomposed to the same extent (not significantly different in Sidak’s multiple comparisons test, α = 0.05). However, the decomposition of **1** in Ham’s F12 cell culture medium was significantly stronger compared to **2** (*p* ≤ 0.001) and **3** (*p* ≤ 0.01). The different stability of the enantiomers **1** and **2** in Ham’s F12 cell culture medium can be attributed to its numerous chiral constituents. Enantioselectivity in the presence of chiral amino acids is frequently observed in organic reactions; still, an unequivocal mechanism often remains obscure [20]. For the potential application as a carbon-11 PET tracer, compounds should exhibit a reasonable stability for at least six half-lifes (i.e., 2 h). The determined stabilities of **1**, **2**, and **3** support further development as a carbon-11 PET tracer, as a linear interpolation between the 0 h and 10 h indicates that only 0.8% and 2.5% are decomposed after 2 h in DPBS and Ham’s F12 cell culture medium, respectively.

### 2.3. In Silico Docking Experiments

**Figure 4:**
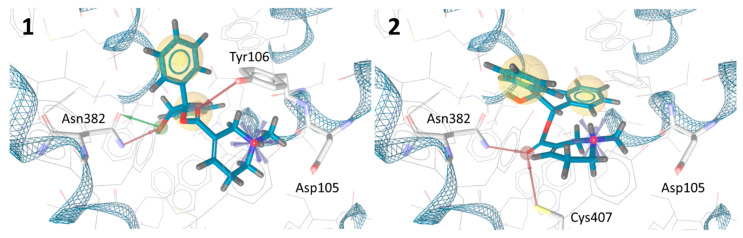
Calculated binding poses of protonated **1** and **2** in the orthosteric binding pocket of human muscarinic acetylcholine receptors (mAChR) M1. Amino acid residues participating in non-hydrophobic interactions are highlighted. **1** shows hydrophobic interactions to Val113, Trp157, Leu183, Ala193, Ala196, Trp378 and **2** to Tyr106, Trp157, Ala193, Ala196, Trp378, Tyr381, and Val385 (not highlighted).

Based on the in silico docking experiments, the enantiomers **1** and **2** feature substantially different binding poses (Figure 4). In addition to the hydrophobic interactions and the ionic Asp105 interaction, both enantiomers show hydrogen bonds to two amino acid side chains. However, in the predicted binding pose of **1**, the position of the hydroxyl group enables it to act as a hydrogen bond donor and acceptor to Asn382, which is an amino acid that is known to be strongly involved in the binding of high affinity antagonists [21]. Conclusively, **1** exhibits an additional hydrogen bond interaction compared to **2**, which can be seen as an indicator for higher affinity.

### 2.4. Biological Evaluation

The expected orthosteric binding of **1**, **2**, and **3** could be confirmed using [*N*-methyl-^3^H]scopolamine methyl chloride ([^3^H]NMS) in a competitive radioligand binding assay toward all mAChR subtypes. All competitive binding curves show a monophasic logistic behavior and justify the use of a one-site model (supporting information, Figure S1) [22]. Comparing the subtypes, all tested compounds show the highest affinity toward M1 followed by M5 and the lowest affinity to M2 (Table 1). However, the methyl ester of the free acid of **1**, **2**, and **3** (**e**, arecoline) shows pronounced mAChR M2 affinity [13], which illustrates the influence of chemical derivatization on subtype selectivity. **1** is considered most promising because it displays the highest affinity to the preferred M1 subtype. Furthermore, **1** shows the strongest subtype selectivity against all subtypes (M1 over M2, M3, M4, and M5) and the highest overall selectivity between M1 and M2 (19-fold) within the tested compounds. Especially, the 7-fold M1 over M4 subtype selectivity has to be mentioned. Although it is the second lowest selectivity compared to the other receptor subtypes, it is even higher than found in pirenzepine (3.5-fold) and trihexyphenidyl (1.6-fold) [23]. The superior mAChR M1 affinity of the (*R*,*R*)-isomer **1** over the (*S*,*S*)-isomer **2** corresponds well to the in silico docking experiments.

**Figure 5:**
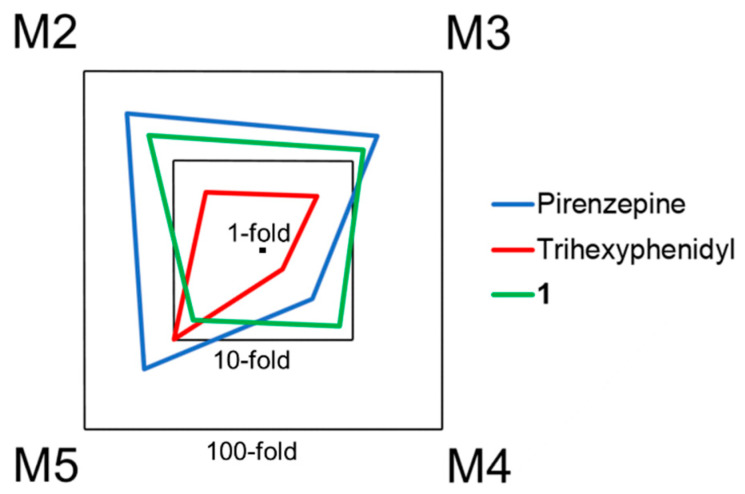
Subtype selectivities related to M1 visualized as radar charts. For each substance, the logarithmic axis represents K_i_ toward the respective subtype divide by the K_i_ of M1. Compared to the clinically established M1 antagonists pirenzepine and trihexyphenidyl, **1** shows a pronounced subtype selectivity toward M4, which results in overall broader subtype selectivity. Selectivities of pirenzepine and trihexyphenidyl were calculated based on their literature reported K_i_ values [23].

In terms of mAChR M1 minimum subtype selectivity, **1** performs better than the clinically established mAChR M1 antagonists pirenzepine and trihexyphenidyl (Figure 5) and also better than our recent top candidates for mAChR M1 PET imaging [13]. Given the lower affinity of **1** compared to pirenzepine, antagonistic mAChR M1 treatment with **1** would require a higher dose, which might be well tolerated due to the broader M1 subtype selectivity. Compounds featuring two aromatic rings are very prominent in mAChR drug development. However, to the best of our knowledge, the hydrobenzoin scaffold has only been utilized in a single publication on muscarinic ligand development [24]. In that publication, compounds 18a–c contain the hydrobenzoin scaffold integrated in a 1,4-dioxane ring and also show the highest affinity toward subtype M1. Still, subtype selectivity is narrower compared to the compounds presented herein.

The significantly different binding affinities observed between the three stereoisomers underline the general possibility to increase mAChR affinity and subtype selectivity of arecaidine esters via stereochemical variations. In view of the vast majority of achiral small molecules, which have been studied as potential mAChR PET tracers [10], we believe that stereochemistry is a useful yet underappreciated tool to optimize compound properties.

**Figure 6:**
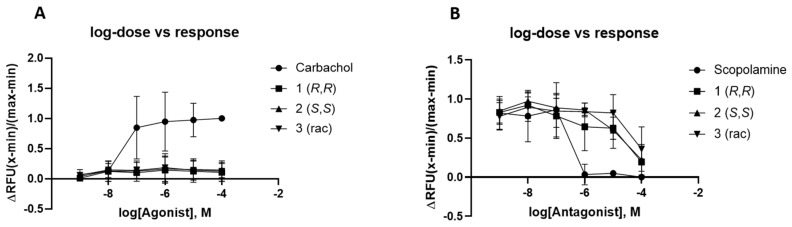
Dose-dependent calcium response to muscarinic 1 (M1) receptor agonists and antagonists. CHO-M1 cells were plated in black clear bottom 96-well plate and incubated overnight. The following day, cells were assayed for a calcium response to carbachol (positive control), **1**, **2**, and **3** using the Fluo-4 Direct™ Calcium Assay Kit. Cells were stimulated for potential agonistic activity (panel A) [27] or cells were treated for potential antagonistic activity, scopolamine (positive control), **1**, **2**, and **3** to block the calcium response elicited by 20 µM carbachol (panel B). Measurements are given in relative fluorescent units (RFU) as the maximum response minus the minimum response divided by the minimum response.

In addition to the assessment of the affinity by means of a radiometric competition binding assay [25] on cellular membranes, the functionality of the synthesized compounds was further evaluated in a cell-based fluorescence assay using Fluo-4 [26]. In contrast to the mAChR agonist carbachol, no increase in calcium release could be induced by incubation with **1**, **2**, and **3** using commercially available Fluo-4 direct calcium flux assay on stably transfected CHO-M1 cells (Figure 6, Panel A). Applying compounds **1**, **2**, **3**, and scopolamine as positive control prior to the treatment with a constant concentration of the signal inducing carbachol clearly illustrates the antagonistic binding of the tested compounds (Figure 6, Panel B). The progression of the effect–concentration curve of both positive controls, carbachol for the agonistic assay and scopolamine for the antagonistic assay, are in good accordance with the results depicted by the manufacturer.

**1** has a high binding affinity (K_i_-value) to the M1 receptor of 99 ± 19 nM, as assessed by means of the radioligand binding assay, being 2-fold out of the postulated ideal range [10]. As numerous mAChR ligands failed in PET radiotracer development due to too high binding affinity, being a disadvantage in radiotracer kinetics causing flow dependence of tracer accumulation [10], this “borderline” binding affinity should not be a common ground for excluding **1** from further development toward diagnostic application, especially when considering that the targeted mAChR subtype M1 is the most abundant subtype in the cortex [28]. Furthermore, **1** may serve as a lead structure for the development of derivatives with even higher affinity, retaining its suitable selectivity profile and moderate lipophilicity values.

It is an ongoing debate as to whether PET radiotracer targeting GPCRs are preferably agonist or antagonists [29] Agonists per se complicate the PET image analysis as they favorably bind to the activated form of GPCRs. The global picture shows a prevalence for antagonists carried by the easier access and unlikeliness of side effects. Considering the physico-chemical parameters, binding affinities, subtype selectivity, and effective functionality of the here presented subset of compounds, the potential application points into the direction as diagnostic imaging probes over a potential therapeutic application. Potential application as an imaging probe is further supported by the expected ease of carbon-11 radiolabeling of **1**, considering that structurally related arecaidine esters were previously labeled with carbon-11 straightforwardly via reaction of [^11^C]CH_3_I with *N*-desmethyl precursors [13]. The observed antagonism is advantageous for PET radiotracers, as potential side effects are unlikely. Especially, compound **1** stands out with binding affinities in the nanomolar range and high subtype selectivity, proving the importance of stereochemical adjustments for successful mAChR ligand development.

## 3. Conclusions

Subtype selectivity represents an ambitious but necessary goal in the development of orthosteric mAChR ligands for therapeutics as well as for diagnostics. Within this work, we synthesized chiral hydrobenzoin esters of arecaidine and investigated their stability and binding properties toward mAChRs. Stereochemistry was shown to have a strong impact on in vitro mAChR M1 affinity, which was also anticipated by in silico molecular docking. Furthermore, the variable affinity toward the other subtypes highlights the relevance of chiral ligands in the quest for subtype selectivity. The antagonistic (*R*,*R*)-isomer **1** shows most promising characteristics to act as a suitable mAChR M1 PET tracer and especially stands out because of its broad subtype selectivity compared to clinically established mAChR M1 antagonists. These properties motivate us to radiolabel **1** with carbon-11 in the future to further evaluate its applicability as brain PET tracer.

## 4. Experimental

### 4.1. Materials

Arecaidine was prepared according to the literature [30] from arecoline hydrobromide (XA BC-Biotech). Carbonyldiimidazol (CDI, reagent grade, Sigma Aldrich, St. Louis, MO, USA), (*R*,*R*)-hydrobenzoin (>99%, TCI), (*S*,*S*)-hydrobenzoin (98+%, alfa aesar), *meso*-hydrobenzoin (>98%, TCI), KO^t^Bu (98%, Sigma Aldrich), dimethylformamide (DMF, 99.8% extra-dry over molecular sieve, Acros), toluene (99.8% anhydrous, Sigma Aldrich, St. Louis, MO, USA), probenecid (>98%, TCI), and DPBS (Gibco, Life Technologies Limited) were used without further purification. Protease Inhibitor Cocktail powder (P2714-1BTL, Sigma-Aldrich, St. Louis, MO, USA) was dissolved in 10 mL water and used as such.

### 4.2. Instrumentation

NMR samples were measured in deuterated chloroform (CDCl_3_, ≥99.8%, stabilized with silver foil, Sigma Aldrich) at 25 °C on a Bruker Avance III 400 spectrometer (400 MHz for ^1^H and 100 MHz for ^13^C). An assignment of NMR signals was achieved by a combination of standard NMR techniques, such as COSY, NOESY, APT, HSQC, and HMBC experiments. The residual CHCl_3_ signal was set to 7.26 ppm for ^1^H spectra and 77.0 ppm for ^13^C spectra. Coupling constants are given in Hz. Mass spectra were obtained on a Bruker maXis 4G instrument (ESI-TOF, HR-MS). Optical rotation was determined in CHCl_3_ using a Schmidt + Haensch UniPol L2000 polarimeter with a 1dm cell. An Agilent HPLC system consisting of an autosampler (series 1100), pump (series 1200) and diode array detector (series 1100) was used for the determination of logP using an apHERA (5 µm, 10 × 6 mm) stationary phase [15]. Compounds **1**–**3** were shown to exceed a purity of 95% by gradient run using the logP HPLC method without the addition of toluene and triphenylene and by isocratic runs on an XSelect^TM^ (HSS T3, 3.5 µm, 100 × 4.6 mm) stationary phase at a flow of 1 mL/min (supporting information, Figure S2). Fluorescence measurements were performed on a BioTek Synergy HTX multi-mode reader. A M-36 tygon tubed Cell Harvester (Brandel^®^) and Whatman™ GF/B filters were used for the filtration of radioligand binding assays. Filter disks were counted with 2 mL Ultima Gold™ (high flashpoint LSC cocktail, PerkinElmer) using a 300 SL Automatic TDCR liquid Scintillation Counter (HIDEX).

### 4.3. Stability Measurements in DPBS and Cell Culture Media

First, 1089 µL DPBS or Ham’s F12 nutrient mixture were mixed with 11 µL of 10 mM stock solution of **1**, **2**, or **3** in DMSO (final DMSO content: 1%) and agitated at 37 °C. Then, 80 µL of the solution was analyzed by UV-HPLC (216 nm) using an XSelect™ (HSS T3, 3.5 μm, 100 × 4.6 mm) stationary phase and 40% acetonitrile in 25 mM (NH_4_)H_2_PO_4_ buffer (pH 9.3) at a flow of 1 mL/min (k**_1_**_,**2**_ = 5.6, k**_3_** = 5.9). The fraction of stable compounds was calculated as the area under the curve of the compound peak for each combination of compound and matrix related to the 0 h time point with 100%. Three non-consecutive HPLC analyses were performed for each time point, combination of compounds, and matrix.

### 4.4. Molecular Docking Studies

Molecular docking was performed on a crystal structure of human mAChR M1 [31] using AutoDock 4.2 with default settings in LigandScout 4.4_RC6. **1** and **2** were docked as protonated tertiary amines to consider the expected species under physiological conditions. A pharmacophore of the binding poses exhibiting the expected ionic interaction to Asp105 was created to visualize proposed interactions of **1** and **2** to amino acid side chains.

### 4.5. Cell Culture

CHO-K1 cells stably transfected with human muscarinic receptors M1–M5 were obtained from The Missouri University of Science and Technology cDNA Resource Center (Cell Catalog#: CEM1000000, CEM2000000, CEM3000000, CEM4000000, CEM5000000) and cultivated in Ham’s F12 Nutrient Mixture (Gibco, Life Technologies Limited, Waltham, MA, USA) containing 10% FBS (Gibco, Life Technologies Limited), 250 µg/mL Geneticin^®^ (G418, Thermo Fisher, Waltham, MA, USA) at 37 °C and 5% CO_2_ in a cell incubator. Gibco^TM^ Trypsin-EDTA (0.05%) was used for passaging cells. Untransfected CHO-K1 cells were obtained from ATCC and cultivated as described above but in media free of Geneticin^®^. Powdered protease inhibitor cocktail (P2714-1BTL, Sigma Aldrich, St. Louis, MO, USA) was dissolved in 10 mL water and used as such.

### 4.6. Affinity Assay Toward mAChRs M1-M5 on CHO Membranes

Cell membranes bearing human mAChRs M1-M5 were prepared as described previously [13]. In short, stably transfected CHO cells were grown to confluence in T175 flasks, washed with ice-cold DPBS, and suspended in 2 mL 10 mM Tris/HCl, 1 mM EDTA-buffer (pH 7.4), and 200 µL protease inhibitor using a cell scraper. A cell homogenate was prepared by passing the cell suspension through a G29 needle and subsequently centrifuged (10 min, 1000× *g*, 4 °C). Ultracentrifugation of the supernatant (30 min, 100,000× *g*, 4 °C) yielded a membrane pellet, which was suspended in 125 µL 50 mM Tris/HCl-buffer (pH 7.4) per T175 flask and stored at −80 °C. Ten confluent T175 flask were processed for one batch.

Inhibition constants (K_i_) were determined with a competitive radioligand binding assay using 50 mM Tris/HCl, 10 mM MgCl_2_, and 1 mM EDTA (pH 7.4) as assay buffer. Then, 5 µL of test compound in DMSO, 50 µL of [*N*-methyl-^3^H]scopolamine methyl chloride ([^3^H]NMS) in assay buffer, and 445 μL of membrane suspension diluted in assay buffer were incubated for 90 min at 23 °C. The membrane-bound activity was recovered by filtration through GF/B filter paper pre-soaked in 0.1 % PEI and measured with a liquid scintillation counter. Maximum binding was measured by using 5 µL plain DMSO, and nonspecific binding was measured by using 5 µL 1 µM scopolamine in DMSO. A final concentration of [^3^H]NMS was 0.2 nM, 0.3 nM, 0.8 nM, 0.2 nM, and 1 nM for M1–M5. Then, 3–12 µL membrane suspension were used per tube. IC_50_ values were calculated by a variable slope logistic regression using at least five distinct concentrations of test compound, pipetted in triplicates. K_i_ values were calculated using the Cheng–Prusoff equation and following K_D_ values of [^3^H]NMS for M1-M5 [13]: 0.18 nM, 0.24 nM, 0.23 nM, 0.10 nM, and 0.35 nM.

### 4.7. Calcium Efflux Assay for Agonist/Antagonists Discrimination

For the Fluo-4 direct^TM^ calcium assay kit (invitrogen), 100 µL of a 5 × 10^5^ cells/mL suspension of CHO-M1 cells were seeded in black clear bottom 96-well plates (Corning). After settling of the cells for 24 h, the kit was performed according to the supplier’s recommendation. In detail, the media was removed, and 50 µL of Hanks-balanced salt solution (HBSS; Sigma Aldrich) were added, followed by 50 µL of the Fluo-4 buffer solution (including probenecid). The 96-well plates were incubated for 60 min at 37 °C in the dark. For the agonist assay, 100 µL of a double-concentrated dilution series of carbachol (positive control), **1**, **2**, and **3** were added with the end concentration of 100, 10, 1, 0.1, 0.01 and 0.001 µM in duplicates. The relative fluorescence was measured with an excitation wavelength of 494 nm and an emission wavelength of 516 nm. For the antagonist assay, 50 µL of a 4-fold concentrated dilution series of scopolamine hydrochloride (positive control), **1**, **2**, and **3** were added in duplicates. Subsequently a 40 µM stock solution of carbachol was added to all wells, and the relative fluorescence was measured with an excitation wavelength of 494 nm and an emission wavelength of 516 nm. Stock solution of **1**, **2**, and **3** were in DMSO with a final concentration of at most 1% of DMSO. All experiments were performed at least in three repetitions.

### 4.8. Calculations and Statistics

If not indicated otherwise, all values are depicted as mean ± standard deviation. All quantitative results were analysed by means of MS Excel^®^ 2013. Significance was tested with GraphPad Prims 6 (version 6.07, San Diego, CA, USA).

### 4.9. Synthesis

*(1R,2R)-(+)-2-hydroxy-1,2-diphenylethyl 1-methyl-1,2,5,6-tetrahydropyridine-3-carboxylate* (**1**). Arecaidine (40.0 mg, 0.284 mmol, 1.0 equiv) was suspended in dry DMF (5 mL) and heated gently until almost everything dissolved. CDI (46.1 mg, 0.284 mmol, 1.0 equiv) was added as solid. After 1 h (*R*,*R*)-hydrobenzoin (122 mg, 0.568 mmol, 2.0 equiv) was added, and the reaction mixture was stirred for 7 days at 20 °C. The reaction mixture was poured on water (15 mL) and extracted with EtOAc (3 × 15 mL). The combined organic phases were dried over Na_2_SO_4_ and concentrated under reduced pressure. The resulting residue was purified via preparative thin layer chromatography (TLC) (CH_2_Cl_2_/MeOH, 90:10) to give the desired product in 13% yield (12.2 mg). ^1^H NMR (400 MHz, CDCl_3_): 7.24–7.17 (m, 6H, Ph H-3,4,5), 7.15–7.07 (m, 5H, Ph H-2,6, H-4), 5.92 (d, *J* = 7.2, 1H, Et H-1), 4.95 (d, *J* = 7.2, 1H, Et H-2), 3.20–3.10 (m, 2H, H-2), 2.53–2.47 (m, 2H, H-6), 2.43–2.35 (m, 2H, H-5), 2.39 (s, 3H, CH_3_). ^13^C NMR (101 MHz, CDCl_3_): δ 164.8 (C=O), 139.2 (Ph C-1), 138.4 (C-4), 136.9 (Ph C-1), 128.8 (C-3), 128.10 (Ph C-3,4,5), 128.03 (Ph C-3,5), 127.97 (Ph C-4), 127.2 (Ph-C-2,6), 127.1 (Ph C-2,6), 79.9 (Et C-1), 77.2 (Et C-2), 53.1 (C-2), 50.6 (C-6), 45.6 (CH_3_), 26.6 (C-5). HRMS (ESI+) (m/z): calculated for C_21_H_24_NO_3_ [M + H]^+^: 338.1751; found 338.1751.

*(1S,2S)-(−)-2-hydroxy-1,2-diphenylethyl 1-methyl-1,2,5,6-tetrahydropyridine-3-carboxylate* (**2**). Following a modified literature procedure [32], arecoline hydrobromide (200 mg, 0.85 mmol, 1.0 equiv) was dissolved in sat. aq. Na_2_CO_3_ (5 mL). The aqueous layer was extracted with CH_2_Cl_2_ (3×), dried (Na_2_SO_4_), and concentrated under reduced pressure. To a solution of the residual oily free base in PhMe (5 mL), (*S*,*S*)-hydrobenzoin (181 mg, 0.85 mmol, 1.0 equiv), KO^t^Bu (5 mg, 0.04 mmol, 0.05 equiv), and a spatula of activated 5 Å molecular sieves were added subsequently. The resulting mixture was refluxed for 16 h. Then, it was filtered through a pad of celite, and volatiles were removed under reduced pressure. The crude oil was purified via preparative TLC (EtOAc/*n*-heptane, 70:30 with 3% Et_3_N) to give the desired product in 22% yield (63 mg). ^1^H NMR (400 MHz, CDCl_3_): δ 7.25–7.19 (m, 6H, Ph H-3,4,5), 7.17–7.06 (m, 5H, Ph H-2,6, H-4), 5.92 (d, *J* = 7.2, 1H, Et H-1), 4.97 (d, *J* = 7.2, 1H, Et H-2), 3.20–3.13 (m, 2H, H-2), 2.53–2.47 (m, 2H, H-6), 2.43–2.36 (m, 2H, H-5), 2.41 (s, 3H, CH_3_). ^13^C NMR (101 MHz, CDCl_3_): δ 164.8 (C=O), 139.0 (Ph C-1), 138.4 (C-4), 136.8 (Ph C-1), 128.9 (C-3), 128.17 (Ph C-4), 128.15 (Ph C-3,5), 128.09 (Ph C-3,5), 128.05 (Ph C-4), 127.2 (Ph C-2,6), 127.1 (Ph C-2,6), 79.9 (Et C-1), 77.2 (Et C-2), 53.2 (C-2), 50.7 (C-6), 45.7 (CH_3_), 26.7 (C-5). HRMS (ESI+) (m/z): calculated for C_21_H_24_NO_3_ [M + H]^+^: 338.1751; found 338.1758.

*Rac. (1S,2R)-2-hydroxy-1,2-diphenylethyl 1-methyl-1,2,5,6-tetrahydropyridine-3-carboxylate* (**3**). Following a modified literature procedure [32], arecoline hydrobromide (200 mg, 0.85 mmol, 1.0 equiv) was dissolved in sat. aq. Na_2_CO_3_ (5 mL). The aqueous layer was extracted with CH_2_Cl_2_ (3×), dried (Na_2_SO_4_), and concentrated under reduced pressure. To a solution of the residual oily free base in PhMe (5 mL), *meso*-hydrobezoin (181 mg, 0.85 mmol, 1.0 equiv), KO^t^Bu (5 mg, 0.04 mmol, 0.05 equiv), and a spatula of activated 5 Å molecular sieves were added subsequently. The resulting mixture was refluxed for 16 h. Then, it was filtered through a pad of celite, and volatiles were removed under reduced pressure. The crude oil was purified via preparative TLC (EtOAc/*n*-heptane, 70:30 with 3% Et_3_N) to give the desired product in 16% yield (46 mg). ^1^H NMR (400 MHz, CDCl_3_): δ 7.33–7.26 (m, 6H, Ph H-3,4,5), 7.26–7.22 (m, 4H, Ph H-2,6), 7.03–6.97 (m, 1H, H-4), 5.97 (d, *J* = 5.8, 1H, Et H-1), 5.03 (d, *J* = 5.8, 1H, Et H-2), 3.13–3.06 (m, 2H, H-2), 2.53–2.45 (m, 2H, H-6), 2.40 (s, 3H, CH_3_), 2.39–2.33 (m, 2H, H-5). ^13^C NMR (101 MHz, CDCl_3_): δ 164.5 (C=O), 139.5 (Ph C-1), 138.2 (C-4), 136.6 (Ph C-1), 128.8 (C-3), 128.38 (Ph C-4), 128.26 (Ph C-3,5), 128.08 (Ph C-3,5, Ph C-4), 78.9 (Et C-1), 76.5 (Et C-2), 53.0 (C-2), 50.6 (C-6), 45.6 (CH_3_), 26.6 (C-5). HRMS (ESI+) (m/z): calculated for C_21_H_24_NO_3_ [M + H]^+^: 338.1751; found 338.1755.

## Figures and Tables

**Table 1:** Inhibition constants (K_i_) given in nM, determined by competition binding on cell membranes derived from Chinese hamster ovary (CHO) cells. For data of this work and [13], errors are reported as standard deviation and for [23], errors are reported as standard error. K_i_ values of this work were determined using [*N*-methyl-^3^H]scopolamine methyl chloride ([^3^H]NMS), *n* ≥ 3.

	M1	M2	M3	M4	M5	Source
**1 (*R*,*R*)**	99 ± 19	1900 ± 300	1300 ± 600	700 ± 300	600 ± 75	this work
**2 (*S*,*S*)**	800 ± 200	8000 ± 2000	1600 ± 300	2700 ± 600	1300 ± 300	this work
**3 (*rac*)**	380 ± 90	3700 ± 1000	3200 ± 500	1600 ± 200	970 ± 90	this work
arecoline	20,300 ± 2400	3800 ± 890	16,700 ± 5900	4700 ± 410	7000 ± 570	[13]
pirenzepine	8 ± 1	270 ± 10	150 ± 10	28 ± 1	170 ± 10	[23]
trihexyphenidyl	1.6 ± 0.2	7 ± 1	6.4 ± 0.4	2.6 ± 0.4	15.9 ± 0.1	[23]

## Supplementary Materials

The following are available online at https://www.mdpi.com/1424-8247/13/12/437/s1, Figure S1: Exemplary competitive binding curves of **1**, **2**, and **3** on human mAChR M1 expressed on cell membranes of stably transfected CHO cells using [*N*-methyl-^3^H]scopolamine methyl chloride. Values at 10^−11^ M and 10^−10^ M represent full binding in absence of competitor, Figure S2: Isocratic HPLC chromatograms of compounds **1**–**3**. 35–40% ACN in 25 mM NH_4_H_2_PO_4_ buffer pH 9.3 at a flow of 1 mL/min, Figure S3: Chiral chromatography using a an AGP 0.3 cmØ × 5cm 5 µM column operated with 2% IPA in 10 mM NH_4_Ac pH 5.8 at a flow of 0.5 mL/min, Figure S4: ^1^H- and ^13^C-NMR spectrum of **1**, Figure S5: ^1^H- and ^13^C-NMR spectrum of **2**. Figure S6: ^1^H- and ^13^C-NMR spectrum of **3**.
