# Supplementary material for: Synthesis, Biological, and Computational Evaluation of Antagonistic, Chiral Hydrobenzoin Esters of Arecaidine Targeting mAChR M1
Source: Pharmaceuticals (Basel). 2020 Nov 30;13(12):437. doi: 10.3390/ph13120437 (PMC7760838; doi:10.3390/ph13120437)
Supplement: Supplementary file 1 [file pharmaceuticals-13-00437-s001.pdf]

pharmaceuticals

Supporting Information

Synthesis, Biological, and Computational Evaluation of Antagonistic, Chiral Hydrobenzoin Esters of Arecaidine Targeting mAChR M1

Marius Ozenil [1], Jonas Aronow [1], Daniela Piljak [1], Chrysoula Vraka [1], Wolfgang Holzer [2], Helmut Spreitzer [2], Wolfgang Wadsak [1,3], Marcus Hacker [1] and Verena Pichler [2,*]

[1] Department of Biomedical Imaging and Image-guided Therapy, Division of Nuclear Medicine, Medical University of Vienna, 1090 Vienna, Austria; marius.ozenil@meduniwien.ac.at (M.O.); jonas.aronow@meduniwien.ac.at (J.A.); daniela.piljak@gmx.at (D.P.); chrysoula.vraka@meduniwien.ac.at (C.V.); wolfgang.wadsak@meduniwien.ac.at (W.W.); marcus.hacker@meduniwien.ac.at (M.H.)
[2] Department of Pharmaceutical Chemistry, Faculty of Life Sciences, University of Vienna, 1090 Vienna, Austria; wolfgang.holzer@univie.ac.at (W.H.); helmut.spreitzer@univie.ac.at (H.S.)
[3] CBmed GmbH - Center for Biomarker Research in Medicine, 8036 Graz, Austria
* Correspondence: verena.pichler@univie.ac.at; Tel.: +43-1-4277-55624

Content:
Exemplary competitive binding curves on human mAChR M1 (Figure S1)
UV-HPLC chromatograms (Figure S2, Figure S3)
NMR spectra (Figure S4, Figure S5, Figure S6)

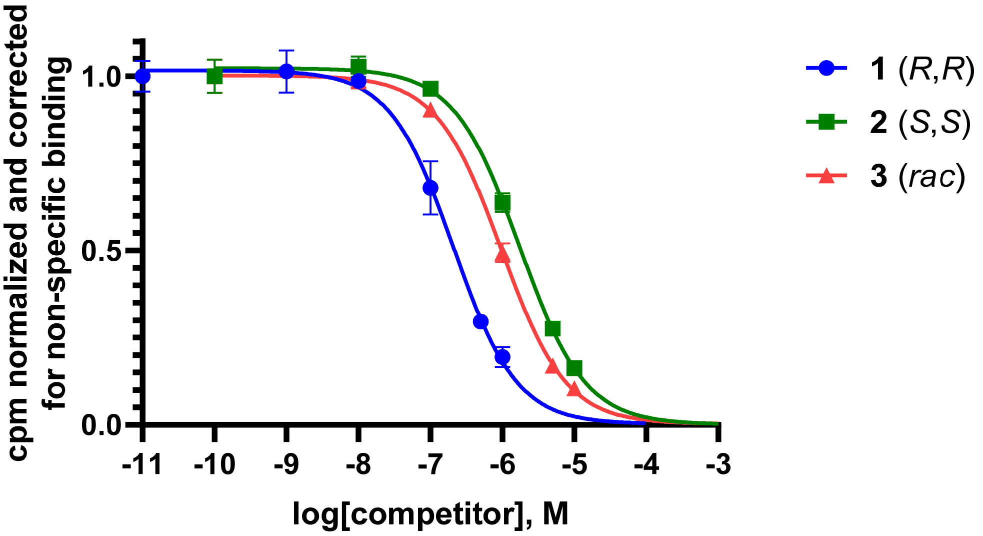

Figure 1. Exemplary competitive binding curves of **1**, **2**, and **3** on human mAChR M1 expressed on cell membranes of stably transfected CHO cells using [*N*-methyl-^{3}H]scopolamine methyl chloride. Values at 10^{-11} M and 10^{-10} M represent full binding in absence of competitor.

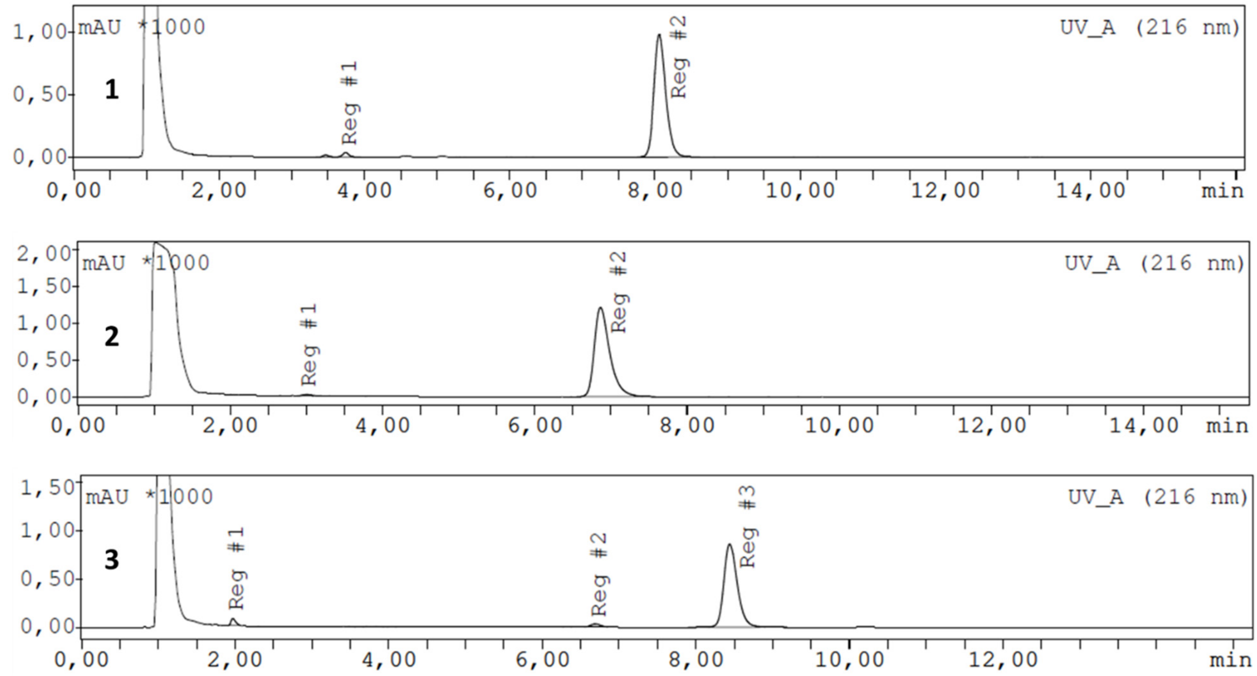

Figure 2. Isocratic HPLC chromatograms of compounds **1**–**3**. 35–40% ACN in 25 mM $NH_4H_2PO_4$ buffer pH 9.3 at a flow of 1 mL/min.

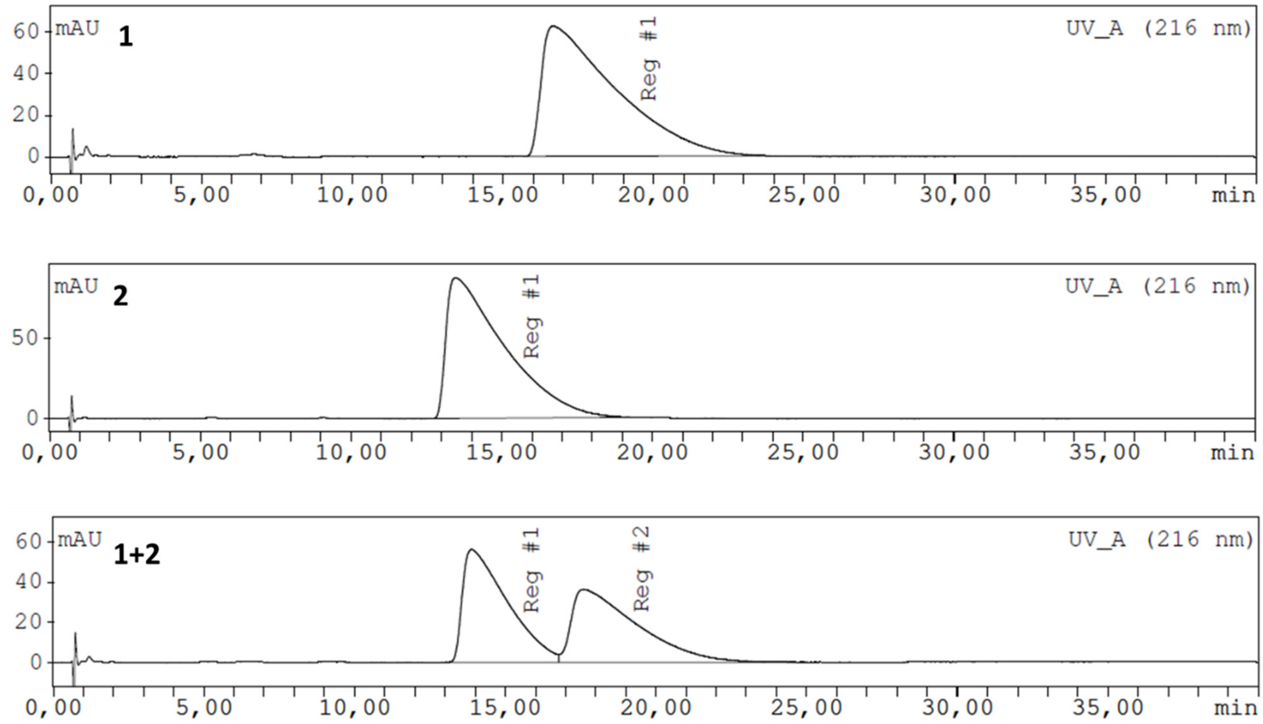

Figure 3. Chiral chromatography using a an AGP 0.3 cmØ × 5 cm 5 μM column operated with 2% IPA in 10 mM NH_4Ac pH 5.8 at a flow of 0.5 mL/min.

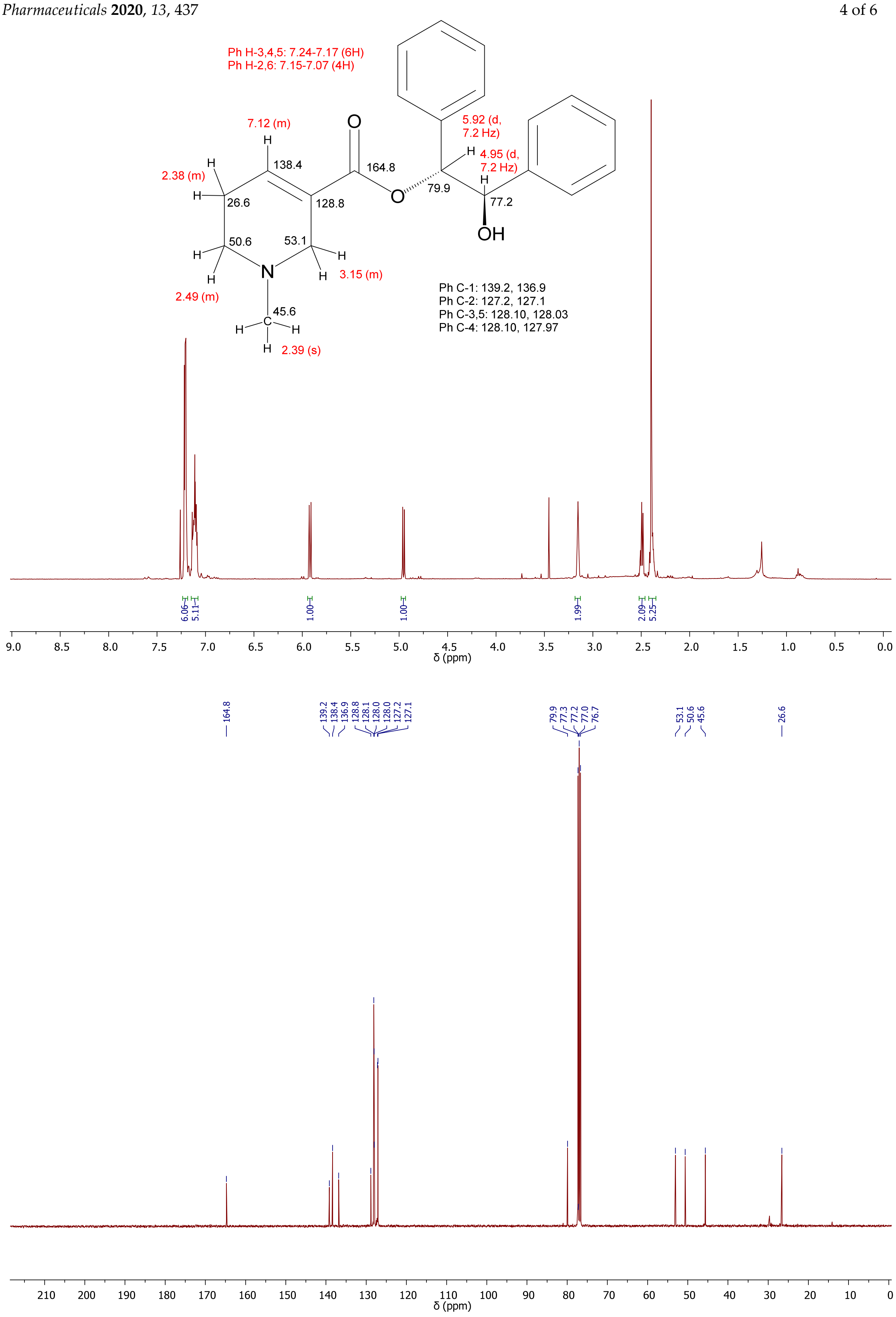

Figure 4. ^{1}H- and ^{13}C-NMR spectrum of **1**.

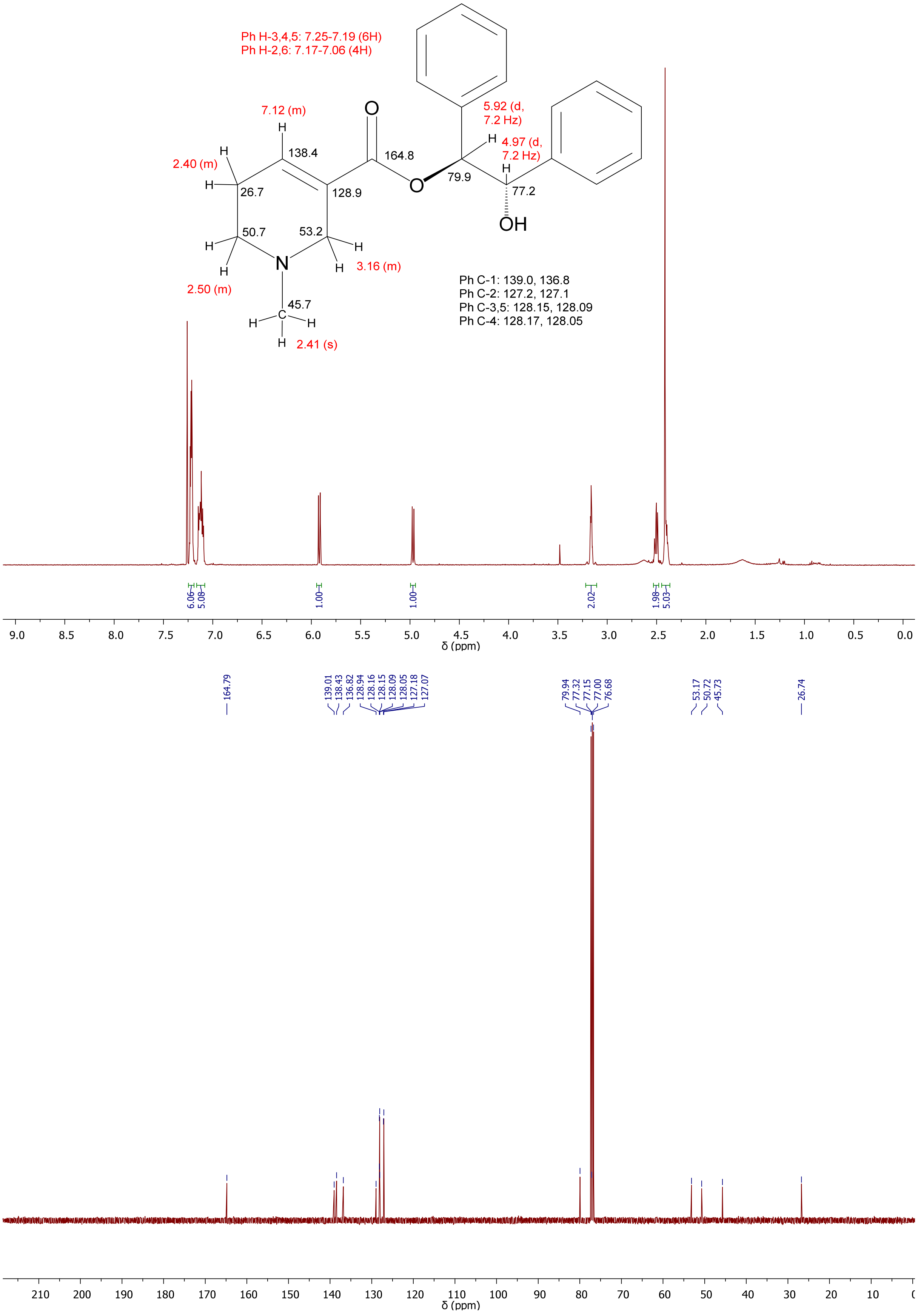

Figure 5. ^{1}H- and ^{13}C-NMR spectrum of **2**.

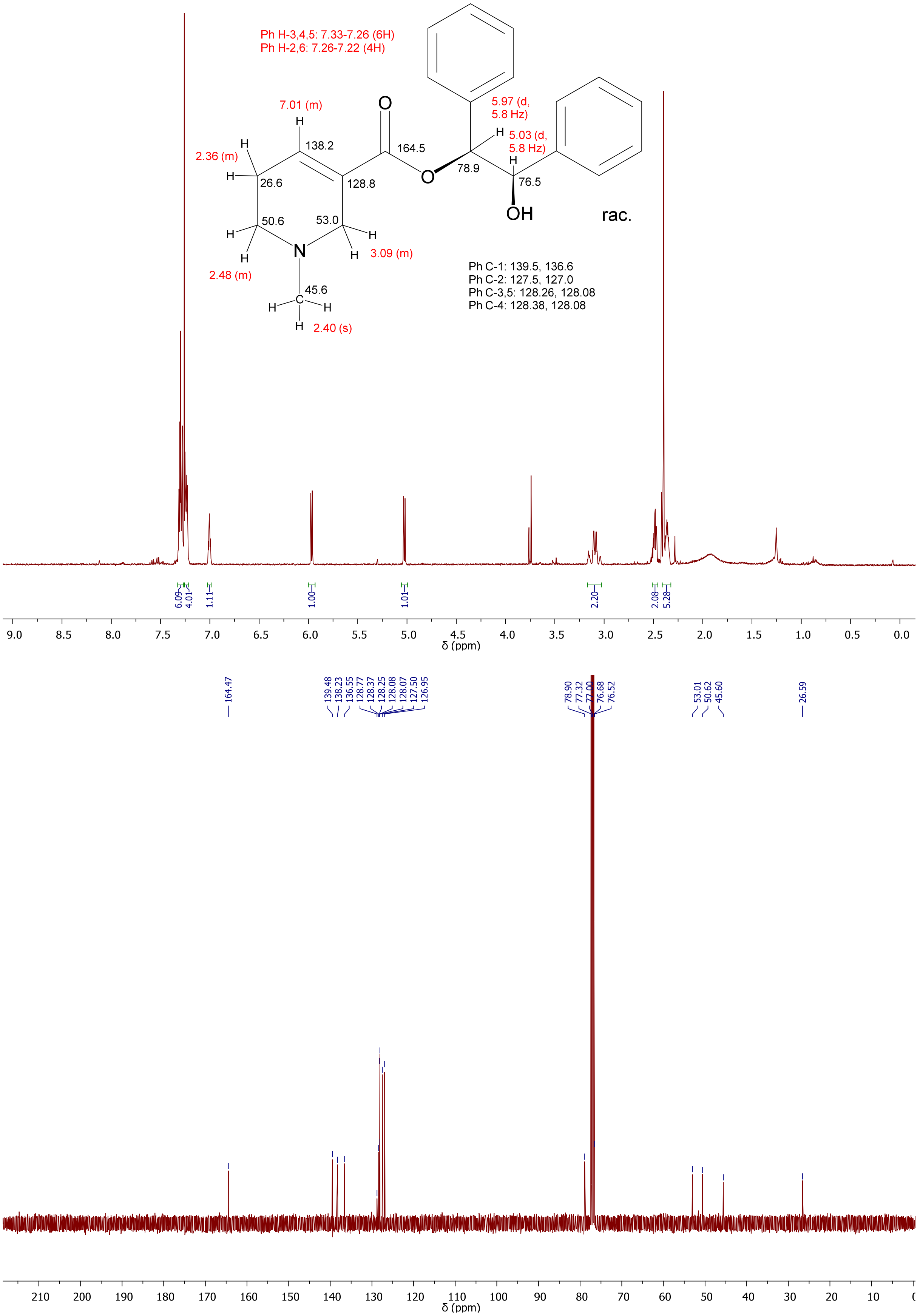

Figure 6. ^{1}H- and ^{13}C-NMR spectrum of **3**.
